# Laparoscopic Treatment of 1522 Adnexal Masses: An 8-Year Experience

**DOI:** 10.1155/2015/979162

**Published:** 2015-02-11

**Authors:** I. Grammatikakis, P. Trompoukis, S. Zervoudis, C. Mavrelos, P. Economides, V. Tziortzioti, N. Evangelinakis, D. Kassanos

**Affiliations:** ^1^3rd Department of Obstetrics and Gynecology, Medical School of Athens, General University Hospital “Attikon”, Greece; ^2^“Lito” Maternity Hospital, Athens, Greece

## Abstract

*Objective*. To reevaluate the long-term effectiveness and safety of laparoscopy in benign ovarian pathology. *Materials and Methods*. 1522 women with benign adnexal cysts, laparoscopically treated in the 3rd Department of Ob/Gyn, General University hospital “Attikon” and “Lito” Maternity Hospital between July 1998 and December 2006, were included. *Results*. The diagnosis in 1222 (80,6%) cases was endometriosis of the ovary, 60 (4%) hydrosalpinx, 51 (3,3%) serous cystadenomas, 44 (2,9%) dermoid ovarian cyst, 38 (2,5%) borderline tumors, 35 (2,3%) unruptured follicles, and 33 (2,2%) paraovarian cysts. In 174 cases (11,5%) laparoscopy was converted to laparotomy due to technical difficulties or suspicion of cancer. In particular, laparotomy was performed in 119 (8%) women due to severe adhesions and 18 (1,2%) women due to bleeding that could not be controlled safely by laparoscopy. In 36 (2,4%) women frozen section during operation revealed malignancy and laparoscopy was converted to laparotomy. A few operative complications were recorded like post-op fever, small hematomas at the trocar entries.* Conclusions*. Laparoscopic surgery seems to offer significant advantages such as reduced hospital stay, less adverse effects, better quality of life, and superior vision especially on surgical treatment of cases like endometriosis.

## 1. Introduction

Benign ovarian pathology remains a significant disorder in women who are in reproductive age in USA and in Europe [1, 2]. During the last decades laparoscopic treatment has been established as a routine method of benign ovarian masses. Adhesion prevention, less operative pain and cosmetic better results are some of the most important advantages of this proceed [3]. However it remains controversial which cases the method is indicated. According to the current evidence the parameters which should be examined preoperatively in order to exclude the possibility of a malignancy are the following: morphology of the ovarian cyst (septations internal bordered, papillary projections, echogenicities, and volume) and characteristics which could be evaluated by using transvaginal ultrasonography. Doppler ultrasonography could also be helpful by examining the mass vascularisation, which increases the possibility of a malignancy. In addition elevated tumor markers like CA 19-9, CA 125, and TATI increase our preoperative diagnostic aims. Purpose of this study was to investigate whether laparoscopy could replace safe and effective surgical treatment of benign ovarian pathology.

## 2. Materials and Methods

The study took place in the 3rd Department of Obstetrics and Gynecology, Medical School of Athens, General University Hospital “Attikon” and “Lito” Maternity Hospital of Athens, between July 2006 and December 2013, including 1522 women with ovarian tumors for which there were no signs preoperatively of malignancy. In particular, including criteria were transvaginal ultrasound with findings suggesting benign ovarian mass, gynecological examination without findings which would suggest malignancy, and preoperative checking of the tumor markers levels especially CA 125 (normal range 0–35 mU/L). Excluding criteria were known contraindications for laparoscopy, such as high BMI or medical reasons, as well as ultrasonographic evidence of malignancy like presentation of septated cysts, papillary projections, low vascular resistance (RI), and pulsativity index (PI). The attended method was briefly the following: after reaching and impacting the peritoneal cavity through a 10 mm subumbilical port, pneumoperitoneum was achieved using carbon dioxide at pressure settings of 15 mmHg. Following this, two ports were incised on the right and left abdomen (5 mm and 10 mm, resp.). Very often a third suprapubic port is used for better dissection in order to avoid healthy ovarian tissue trauma. Curved dissector and scissors were used for dissection most of the times. Bipolar coagulation was used for hemostasis and in the cases coagulation failed, sutures were placed. Sutures were placed in only 27 cases. Our aim is always the excision of the ovarian mass, without rupture, even if in some cases especially in “chocolate” endometriotic cysts it was technically difficult and inevitable.

## 3. Results

The study group consisted of 1522 women with a mean age of 42 years (21–70). In all cases the diagnosis was confirmed histologically. According to this results endometriosis was the most common disease, diagnosed in 1222 (80,6%) cases, whereas the diagnosis of the rest of the masses was as follows: 44 (2,9%) dermoid cysts, 35 (2,3%) unruptured follicles, 51 (3,3%) serous cystadenoma, 60 (4%) hydrosalpinx, 38 (2,5%) borderline tumors, and 33 (2,2%) paraovarian cysts. In 174 cases (11,5%) laparoscopy was converted to laparotomy due to technical difficulties or suspicion of cancer. In particular, laparotomy was performed in 119 (8%) women due to severe adhesions and 18 (1,2%) due to bleeding that could not be controlled safely by laparoscopy. In 36 (2,4%) women frozen section during operation revealed malignancy and laparoscopy was converted to laparotomy. The cyst was removed unruptured in a plastic bag in 68% of the cases, while in the rest 32% it was ruptured in the peritoneum. A few operative complications were recorded like postsurgical fever and small hematoma formation of the abdominal wall at the trocar entries. The women were discharged, normally, one day after surgery. The overall complication rate was 11,3%. Major complications occurred in seven patients (0,6%): in three cases the bowel was injured, whereas the urinary bladder was injured in four cases. The major complications were treated successfully during the operation without the need of second surgery after consulting the surgical and urologic departments. More in detail the bowel was checked and sutured intraoperatively with no need for ileostomy or other resection and anastomosis, and the bladder injuries again sutured without any post-op problems.

## 4. Discussion

Benign ovarian masses consist of one of the most common issues which the gynecologist has to treat. Laparotomy considered to be the indicated treatment method until endoscopic methods has been developed years ago. Since this time the continual development of laparoscopy allowed us today to accept endoscopy as a safe and effective alternative to laparotomy, which has additionally important advantages concerning the patients condition after surgery. Even if this successful treatment tends to be the most frequent used surgical method, there are still debates concerning the safety and the effectiveness especially for the endometriosis cases or suspicious masses. Some authors attribute to laparoscopy abdominal wall metastases in cases of ovarian malignancy [4]. However according to the existing evidence, all reported cases occurred in advanced disease stage, pointing out that the disease's progress was established mainly due to the late diagnosis [4–6]. Furthermore, other mechanisms which are thought to participate in tumor cell metastasis are the use of CO_2_. Laser stimulates proteins and induces an increase of mitotic activity, fibroblast number, synthesis of collagen, and neovascularization [7]. In addition hypoxemia induced by CO_2_ pneumoperitoneum could present a cofactor in adhesion formation. However according to the current data laser lesions do not induce more adhesions than bipolar lesions. The larger denuded area might be important, but what is the main role of depth and amount of tissue necrosis and whether this effect might be specific for hypoxemia induced adhesions should be investigated. Recent publications indicate this could be mediated by a growth factor such as TGF-*β* or VEGF and more specific studies are currently being performed in order to clarify this point.

On the contrary a meta-analysis of related studies [8] comparing laparoscopy to laparotomy in treatment of benign adnexal masses points out the advantages of this proceed. It has been shown that laparoscopy caused less adverse effects, less postoperative pain, shorter stay in hospital, smaller readmission rates, better panoramic vision, and significantly lower cost. It is today almost accepted that laparoscopy is more beneficial than laparotomy but the importance of patient selection and availability of a gynecologic oncologist should be taken into account. Potential malignant tumors should be carefully excluded from laparoscopic options. This is performed by the use of transvaginal ultrasonography combined where possible with Doppler and 3D ultrasound [9]. In that cases the parameters that have to be examined with attention are diameter of the cyst larger than 5 cm, presence of unilocular or bilateral ovarian cysts, existence of septa, and solid particles of papillomatous structures. Concerning the tumor markers CA-125 has a special predictive value in premenopausal women, even though false positive values are expected in cases of endometriosis and uterine fibromas. Lastly, endometriosis and uterine fibromas in postmenopausal women have a certain draw towards malignancy [9].

In our study we showed that in 1522 cases there were 36 (2,36%) malignancies that were revealed during surgery, despite the extensive preoperative investigation. In these cases immediate conversion to laparotomy was conducted and no wall metastasis or other particular adverse effects for the patient were recorded. From our experience ultrasound morphology was significantly more important than preoperative CA-125 in detecting malignancies. It should be emphasized though that in large ovarian cysts (>10 cm) transvaginal ultrasonography might be limited in visualizing the entire cyst contents, so that transabdominal ultrasound and pelvic MRI could have better results [10]. The increased risk for the rupture of the mass and spillage of cyst contents remains the most reported complication providing an important disadvantage of the method. On the contrary, a debate exists there, because according to the current literature it is not clearly reported which is the estimated risk of rupture during laparotomy, since surgeons seldom refer to the risk of rupturing a cyst when it is removed by laparotomy. This suspicion is not supported by several investigators that report the intact cystectomy by laparoscopy in rates up to 80% [11], or similar rates when laparoscopy and laparotomy are compared. Yuen et al. report [12] that the cyst was removed unruptured in 72,2% of the cases, something that is comparable to the 68% of our study. Many surgeons first puncture cysts after putting them in the endobag, but this is not a general rule.

The current study adds our experience in favor of laparoscopy in treatment of a large population of women with benign adnexal masses. Significant advantages such as reduced hospital stay, less adverse effects, and better quality of life are benefits of laparoscopy.

## References

[1] Westhoff C., Clark C. J. G. (1992). Benign ovarian cysts in England and Wales and in the United States. *British Journal of Obstetrics and Gynaecology*.

[2] Hilger W. S., Magrina J. F., Magtibay P. M. (2006). Laparoscopic management of the adnexal mass. *Clinical Obstetrics and Gynecology*.

[3] Canis M., Mage G., Pouly J. L., Wattiez A., Manhes H., Bruhat M. A. (1994). Laparoscopic diagnosis of adnexal cystic masses: a 12-year experience with long-term follow-up. *Obstetrics and Gynecology*.

[4] Matsushita H., Takayanagi T., Ikarashi H., Fukase M. (2013). Ovarian cancer presenting as a metastasis to a trocar tract used for a gasless lift-laparoscopy to resect a benign ovarian cyst: an unusual case report. *European Journal of Gynaecological Oncology*.

[5] van Dam P. A., DeCloedt J., Tjalma W. A. A., Buytaert P., Becquart D., Vergote I. B. (1999). Trocar implantation metastasis after laparoscopy in patients with advanced ovarian cancer: can the risk be reduced?. *American Journal of Obstetrics & Gynecology*.

[6] Leminen A., Lehtovirta P. (1999). Spread of ovarian cancer after laparoscopic surgery: report of eight cases. *Gynecologic Oncology*.

[7] Pinto R. M., Michos G., Papageorgiou G., Halmos G., Moustafa M., Magos A. (2014). Incidence of unplanned oophorectomy at laparoscopic ovarian cystectomy for clinically benign cysts. *Journal of Obstetrics & Gynaecology*.

[8] Medeiros L. R., Stein A. T., Fachel J., Garry R., Furness S. (2008). Laparoscopy versus laparotomy for benign ovarian tumor: a systematic review and meta-analysis. *International Journal of Gynecological Cancer*.

[9] Matsushita H., Watanabe K., Yokoi T., Wakatsuki A. (2014). Unexpected ovarian malignancy following laparoscopic excision of adnexal masses. *Human Reproduction*.

[10] Eltabbakh G. H., Charboneau A. M., Eltabbakh N. G. (2008). Laparoscopic surgery for large benign ovarian cysts. *Gynecologic Oncology*.

[11] Djukic M., Stankovic Z., Vasiljevic M., Savic D., Lukac B., Djuricic S. (2014). Laparoscopic management of ovarian benign masses. *Clinical and Experimental Obstetrics & Gynecology*.

[12] Yuen P. M., Rogers M. S. (1994). Laparoscopic management of ovarian masses: the initial experience and learning curve. *Australian and New Zealand Journal of Obstetrics and Gynaecology*.

